# Synergistic effect of serum uric acid and body mass index trajectories during middle to late childhood on elevation of liver enzymes in early adolescence: Findings from the Ewha Birth and Growth Study

**DOI:** 10.1371/journal.pone.0282830

**Published:** 2023-04-24

**Authors:** Sung Hee Lee, Eun Jeong Choi, Ui Jeong Kim, Hyunjin Park, Bomi Park, Hye Ah Lee, Hyesook Park

**Affiliations:** 1 Department of Preventive Medicine, College of Medicine, Ewha Womans University, Seoul, Korea; 2 Department of Preventive Medicine, Graduate Program in System Health Science and Engineering, College of Medicine, Ewha Womans University, Seoul, Korea; 3 Department of Preventive Medicine, College of Medicine, Chung-Ang University, Seoul, Korea; 4 Clinical Trial Center, Ewha Womans University Mokdong Hospital, Seoul, Korea; Texas A&M University College Station, UNITED STATES

## Abstract

**Background/objectives:**

We aimed to determine whether serum uric acid (SUA) and body mass index (BMI) trajectories in childhood have longitudinal association with liver enzymes in adolescence.

**Methods:**

We conducted a study using data from the Ewha Birth and Growth Cohort. Individual trajectories of **SUA (n = 203)** and BMI (n = 206) from 5, 7, and 9 years were defined by group-based trajectory modeling. Also, liver function enzymes were collected at 11 to 12 year of age (Aspartate Aminotransferase [AST], Alanine transaminase [ALT], and Gamma-glutamyl transferase [γ–GTP]) (n = 206). Using a generalized linear model, the effects of SUA trajectory and BMI trajectory on liver function enzymes were assessed. We also assessed the interaction effect of SUA and BMI trajectories on liver enzymes.

**Results:**

For trajectory patterns, both SUA and BMI were classified into two distinct groups (High or Low). Both trajectory of SUA and BMI in childhood were positively associated with levels of liver enzymes at 11–12 years of age. The results showed that the combined effect of SUA and BMI trajectories on liver enzymes had a higher means in high-risk group (high SUA–high BMI trajectories group) than in low-risk group (low SUA-low BMI trajectories group) for ALT and γ–GTP, respectively. It remained significant association when adjusted for covariates. In addition, the interaction of BMI and SUA trajectories showed a significant synergistic effect.

**Conclusion:**

Elevated childhood SUA and BMI trajectories are associated with increased liver enzymes in beginning of adolescent. This finding suggesting that early interventions in SUA and BMI may need for optimization of liver enzymes as potential marker for development of related disease in later life.

## Introduction

The global epidemic proportions of metabolic syndrome (MetS) was estimated to be around 20–25% [1], and the prevalence of MetS was found to increase substantially in the U.S. according to National Health and Nutrition Examination Survey (NHANES) of 1988–2012 [2]. Using the data from Korea National Health and Nutrition Examination Survey (KNHANES), The overall prevalence of MetS were shown 21.6% in 2007, 19.5% in 2014, and 22.9% in 2018. which has a similar trend with NHANES in US [3]. Also, prevalence of MetS in children and adolescents has been increased statistically significantly. Prevalence trends in MetS were 7.5%, 9.8%, 10.9%, and 6.7% in the KNHANES I through IV (1998–2008) among Korean children and adolescents, respectively (*p*<0.001) [4].

The global prevalence of nonalcoholic fatty liver disease (NAFLD), which is related to metabolic diseases, is estimated to be 25.2% [5]. In a study of adolescents and young adults in the United States, the estimated prevalence of NAFLD was 18.5% [6]. An analysis of Korean National Health and Nutrition Examination Survey data showed that the prevalence of NAFLD in 2016–2017 was 21.5%, compared with 18.6% in 1998–2001 [7], and that the prevalence and severity of NAFLD in childhood and adolescence increased with the prevalence of obesity [8]. Although liver enzyme levels are not direct determinants of MetS, the associations of MetS with metabolic indices and liver enzymes have been investigated [9–11]. A study that used liver enzyme as an indicator of MetS reported a result where prepubertal-stage children with obesity had elevated values of liver enzymes, leptin, markers of insulin resistance, and variables associated with MetS. The result has shown mean of ALT levels were significantly higher in children with obesity compared to in the control group [12].

The SUA level and BMI are reportedly associated with liver enzymes [13, 14]. Overweight and elevated AST and ALT levels were identified as MetS manifestations among children in Korea [15]. Similarly, mean liver enzyme levels at puberty were higher in children with high BMIs than in those with normal BMIs at 3–5 and 7–9 years of age in another study [16]. SUA has already been known through massive studies as a predictor of development of cardiovascular diseases [17, 18], and NHANES in US reported association of SUA with development of chronic liver diseases. SUA level was associated with elevated ALT or γ–GTP, two markers of hepatic necroinflammation [19].

Although associations of liver enzymes with the BMI and SUA level have been reported, no study has evaluated how changes in the BMI or SUA level over time affect liver enzymes.

**Fig 1:**
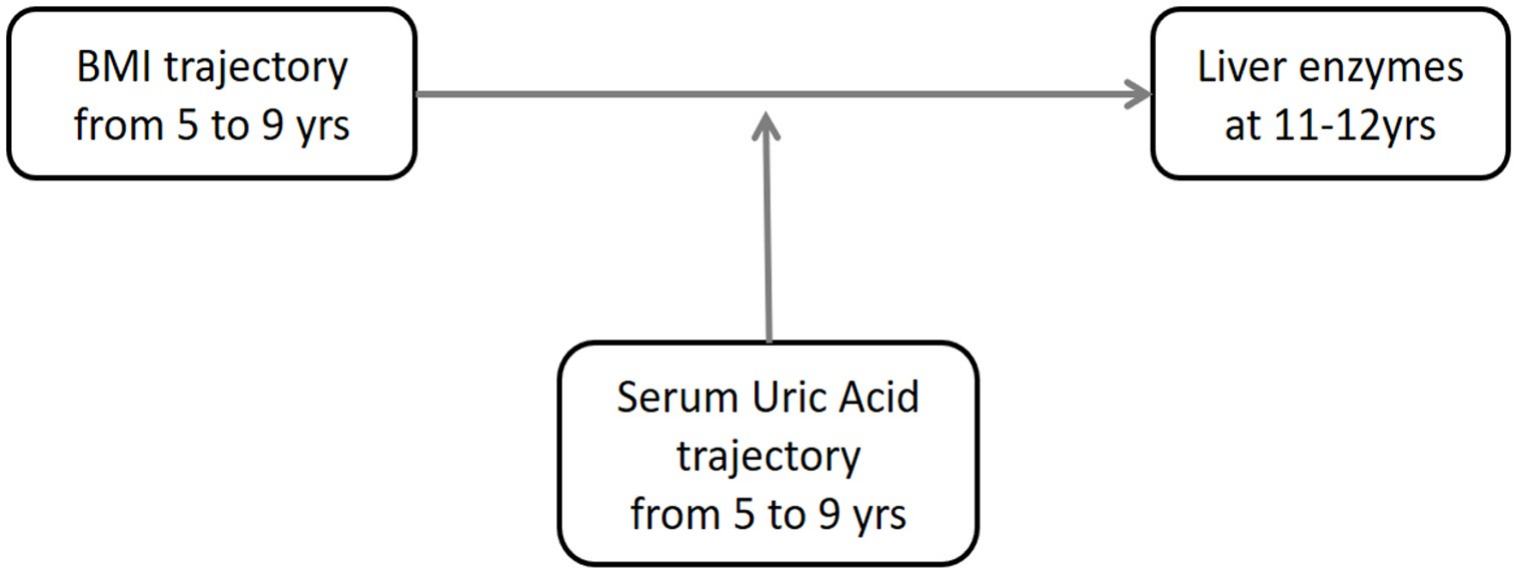
A study hypothesis on the interaction effect of serum uric acid and BMI trajectories during childhood on liver enzymes at 11–12 years of age.

Studies evaluating both factors simultaneously are also lacking. Hence, using a prospective cohort data, we identified trajectories pattern of both SUA and BMI in middle to late childhood (5, 7 and 9 years of age) as risk factors for elevated liver enzymes, and evaluated the combined effect of both trajectories on liver enzymes in early adolescence. We also evaluated the interactive effects of SUA and BMI trajectories on liver enzymes (Fig 1).

## Methods

### Study subjects

This study used data of Ewha Birth and Growth Cohort, which was initiated in 2001 at Ewha Womans University Mokdong Hospital. Details of the birth cohort have been previously reported [20]. Regular check-up was conducted at 3, 5 and 7 years of age and annually thereafter. Of the 939 children enrolled in the birth cohort, data were available on 383 children at 5 years old (197 boys, 186 girls), on 356 children at 7 years old (174 boys, 182 girls), on 400 children at 9 years old (201 boys, 199 girls), and, on 206 at 11 to 12 years old (109 boys, 97 girls) in this analysis.

At regular check-up, blood and urine samples were collected, as well as anthropometric measurements data and data for demographic, socioeconomic, and dietary intake survey using a structured questionnaire. Individual trajectories of SUA and BMI from 5, 7, and 9 years (n = 642) were defined by group-based trajectory modeling, and liver function enzyme were measured at 11 and 12 year of age used in the analysis (n = 206).

### Variables

At the follow-up visits, trained examiners measured current weight to one decimal place with a calibrated scale while the subjects wore light clothing; they measured height in stocking feet using a stadiometer (DS-102 model; Dong Sahn Jenix, Seoul, Korea). BMI was calculated as weight in kilograms divided by height in meters squared (kg/m^2^). The SUA level (mg/dL) was measured using the uricase- and peroxidase-coupled reaction method on a Hitachi 7180 chemistry analyzer (Hitachi, Fukuoka, Japan).

We included appropriate covariates such as sex, age, monthly household income, maternal educational level in this study based on review of literatures [21, 22]. The information for covariates was collected from the questionnaires at 11 to 12 years of age. Monthly household incomes (< 1 million won, 1–2 million won, 2–3 million won, 3–5 million won, and >5 million won) were divided into two categories (<5 million won, and ≥5 million won). Maternal educational levels (elementary/middle school, high school, college, graduate school or higher) were divided into two categories (high school or lower and college or higher).

### Outcomes

We considered liver enzyme level at 11 to 12 years of age as a major outcome; aspartate aminotransferase (AST), alanine aminotransferase (ALT), and gamma-glutamyl transferase (γ–GTP). Serum samples were sent to a diagnostic laboratory (Seegene Medical Institute, Korea) and analyzed using enzymatic methods on a Cobas 8000 C702 autoanalyzer (Roche, Germany). The following substrates (Roche, Germany) were used: ASTL to measure AST activity; ALTL to assess ALT activity; and GGT Gen.2 to assay γ–GTP activity. The coefficient of variation (CV) for all analyses were ≤5%. In this study, the AST, ALT, and γ-GTP levels were distributed normally. The AST/ALT ratio was also calculated.

### Statistical analysis

Continuous data are expressed as the mean ± SD (normally distributed data) and categorical data are expressed as the number of subjects with percentages.

SUA variability was defined as the standard deviations (SD) of Z-scores of SUA across study visits [23]. Trajectories of SUA and BMI at 5,7, and 9 years of age were used as independent factors. Trajectory analysis was conducted using SAS PROC Traj to identify clusters of participants with similar progressions of the SUA level and BMI. The best-fitting model in terms of trajectory number and shape was selected based on the Akaike information criterion (AIC), the Bayesian information criterion (BIC), and group size. Individuals were assigned to trajectory groups based on whether the average posterior probability for that trajectory group was ≥ 0.7 [24, 25].

Using a generalized linear model (GLM), we assessed the effects of SUA and BMI trajectories on liver enzymes and the AST/ALT ratio. We constructed a general linear model after adjusting for sex, age, maternal education level and monthly household income. The results are expressed as adjusted means with 95% confidence interval (95% CI) and a *p*-value. To assess the combined effect of SUA trajectory and BMI trajectory on liver enzymes, individuals were classified based on the combination of SUA and BMI trajectories. The mean difference of liver enzymes according to the combination group of SUA and BMI trajectory was assessed using GLM. Post-hoc test was conducted using the Bonferroni method. We also evaluated the interaction effect and presented the results as *p*-values.

The data were analyzed using SAS ver. 9.4 (SAS Institute, Cary, NC, USA). A two-tailed *p*-value < 0.05 were considered to reflect significance.

### Ethics statement

The parents or guardians of all participants provided written informed consent, and the study protocol was approved by the Institutional Review Board (IRB) of Ewha Womans University Seoul Hospital (number: SEUMC- 2019-04-034). Participants were informed that they could withdraw from the study at any time.

## Results

Table 1 summarizes the basic characteristics of the study subjects. The liver enzymes levels of 206 subjects were followed in adolescent period (11 to 12 years of age). The mean liver enzyme levels were significantly different between boys and girls (*p*<0.01) at 11 to 12 years of age.

**Table 1:** Characteristics of study participants.

	Total		Boys		Girls		*p*-value
	n	Mean ± SD	n	Mean ± SD	n	Mean ± SD	
**5 years of age**							
BMI (kg/m^2^)	383	15.7 ± 1.64	197	15.9 ± 1.75	186	15.5 ± 1.50	0.03
SUA (mg/dL)	310	3.9 ± 0.70	160	3.9 ± 0.65	150	4.0 ± 0.74	0.74
**7 years of age**							
BMI (kg/m^2^)	356	15.9 ± 2.13	174	16.2 ± 2.21	182	15.8 ± 2.03	0.08
SUA (mg/dL)	349	3.7 ± 0.96	169	3.6 ± 0.98	180	3.7 ± 0.93	0.33
**9 years of age**							
BMI (kg/m^2^)	400	17.5±2.61	201	17.8±2.79	199	17.2±2.39	0.02
SUA (mg/dL)	395	3.6±0.84	196	3.6±0.87	199	3.5±0.81	0.50
**11–12 years of age**							
AST (IU/L)	206	23.0±5.72	109	24.4±6.54	97	21.4±4.11	<0.01
ALT (IU/L)	206	14.1±9.03	109	16.0±11.48	97	12.0±4.18	<0.01
γ-GTP (IU/L)	206	14.3±6.45	109	15.7±7.94	97	12.8±3.70	<0.01
**Monthly household income**							
<5 million KRW (n,%)	89 (43.8%)		43 (40.6%)		46 (47.4%)		0.33
≥ 5 million KRW (n,%)	114 (56.2%)		63 (59.4%)		51 (52.6%)		
**Mother’s education level**							
Graduated from high school (n,%)	44 (21.4%)		16 (14.7%)		28 (28.9%)		0.01
Some college or higher (n,%)	162 (78.6%)		93 (85.3%)		69 (71.1%)		

SD, Standard deviation; BMI, Body mass index; SUA, Serum uric acid; AST, aspartate aminotransferase; ALT, alanine aminotransferase; γ-GTP, gamma-glutamyl transferase

**Fig 2:**
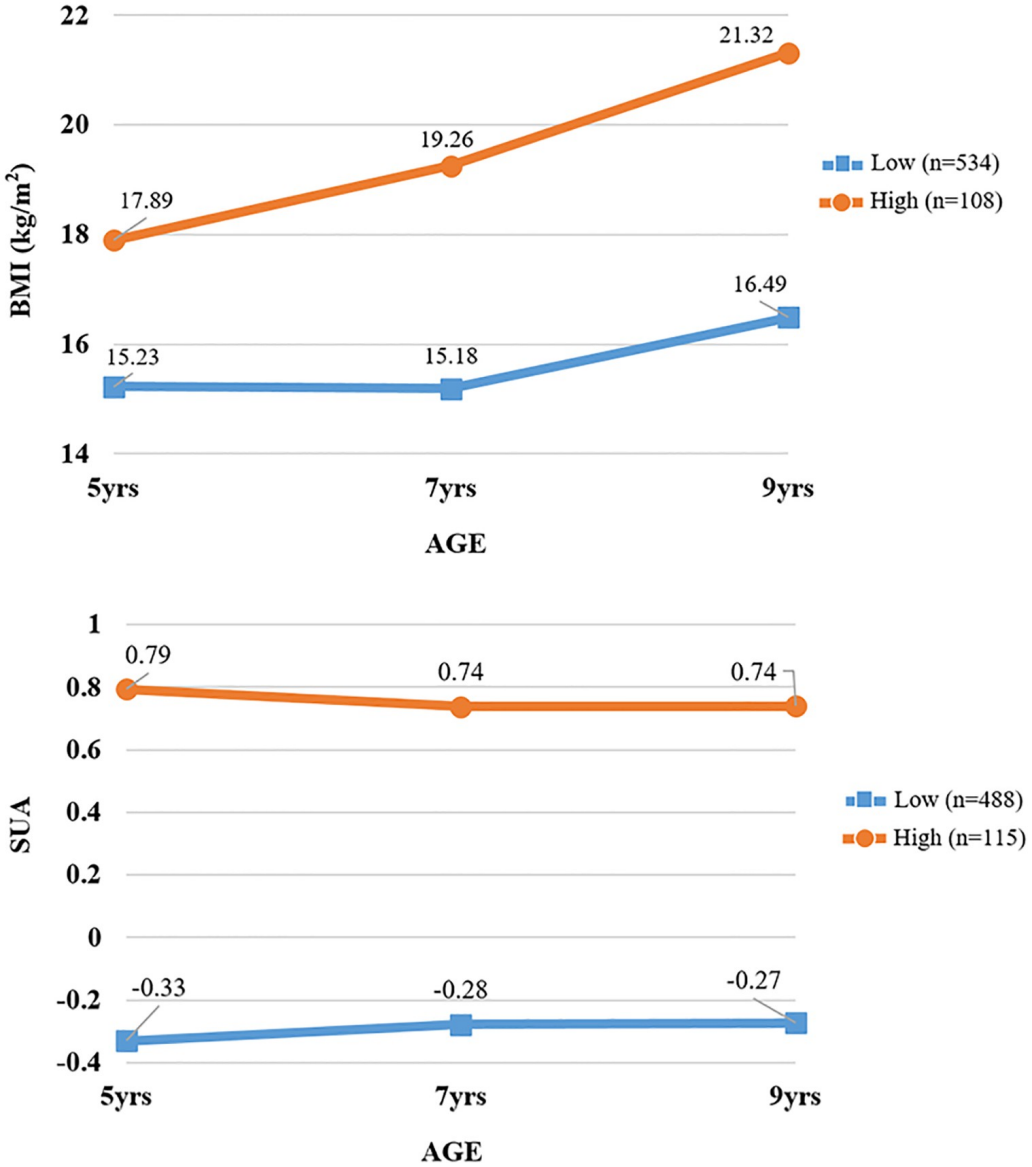
Group-based trajectory modeling used to determine distinct body mass index and serum uric acid trajectories. BMI, Body mass index; SUA, Serum uric acid; * Serum uric acid trajectories were analyzed using standardized serum uric acid levels.

Based on the SUA level and BMI during middle to late childhood, two patterns were identified in these participants, respectively; low and high groups of SUA and BMI, respectively (Fig 2).

The liver enzymes (AST, ALT, and γ–GTP) in our study showed a normal distribution (S1 Fig), and the AST/ALT ratio was also calculated and used as outcome indicator. The effects of SUA and BMI trajectories on liver function enzymes in adolescence have been shown in Tables 2 and 3. In the crude model, high BMI trajectory group had higher mean liver enzymes (AST, ALT, and γ–GTP) than low BMI trajectory group. The mean AST/ALT ratio differed significantly according to the BMI trajectory, and was low in the high BMI group. The high SUA trajectory group had higher mean ALT and γ-GTP levels than did the low SUA trajectory group. The mean AST/ALT ratio was low in the high SUA trajectory group, albeit not significantly so. The significance remained in adjusted model in Table 3 which was adjusted for sex, age, maternal educational level and monthly household income.

**Table 2:** The effects of trajectories on liver function enzyme in adolescent (unadjusted).

	n	AST (IU/L)		ALT (IU/L)		γ-GTP (IU/L)		AST/ALT ratio	
		LS Means	*p*-value	LS Means	*p*-value	LS Means	*p*-value	LS Means	*p*-value
		(95% CI)		(95% CI)		(95% CI)		(95% CI)	
**BMI Trajectory**									
Low	173	22.6	0.04	12.8	<0.01	13.3	<0.01	1.91	<0.01
		(21.8, 23.5)		(11.5, 14.1)		(12.4, 14.2)		(1.84, 1.99)	
High	33	24.8		20.7		19.5		1.50	
		(22.9, 26.8)		(17.8, 23.7)		(17.4, 21.6)		(1.33, 1.67)	
**SUA Trajectory**									
Low	161	22.9	0.85	13.3	0.02	13.8	0.04	1.88	0.09
		(22.0, 23.8)		(11.9, 14.6)		(12.8, 14.8)		(1.80, 1.96)	
High	42	23.1		17.0		16.1		1.72	
		(21.4, 24.8)		(14.3, 19.7)		(14.2, 18.1)		(1.56, 1.88)	
Combined (BMI*SUA) Trajectory									
Low BMI*	140	22.7	0.11	12.7	<0.01	13.3	<0.01	1.92	<0.01
Low SUA		(21.8, 23.7)		(11.3, 14.1)		(12.3, 14.3)		(1.84, 2.00)	
Low BMI*	30	21.9		12.9		13.4		1.87	
High SUA		(19.8, 23.9)		(9.9, 15.9)		(11.2, 15.5)		(1.69, 2.05)	
High BMI*	21	24.1		17.0		17.5		1.59	
Low SUA		(21.7, 26.5)		(13.4, 20.5)		(14.9, 20.1)		(1.37, 1.80)	
High BMI*	12	26.2		27.3		23.1		1.35	
High SUA		(22.9, 29.4)		(22.6, 32.0)^a^^,^^b^^,^^c^		(19.7, 26.5)^a^^,^^b^		(1.07, 1.63)^a^^,^^b^	

LS means, Least-squares means; 95% CI, 95% Confidence interval; BMI, Body mass index; SUA, Serum uric acid; AST, Aspartate aminotransferase; ALT, Alanine aminotransferase; γ-GTP, Gamma-glutamyl transferase.

^a^, Bonferroni-adjusted *p*-value <0.05, compared to the low-low group.

^b^, Bonferroni-adjusted *p*-value <0.05, compared to the low-high group.

^c^, Bonferroni-adjusted *p*-value <0.05, compared to the high-low group.

**Table 3:** The effects of trajectories on liver function enzyme in adolescent (adjusted).

	n	AST (IU/L)		ALT (IU/L)		γ-GTP (IU/L)		AST/ALT ratio	
		LS Means	*p*-value	LS Means	*p*-value	LS Means	*p*-value	LS Means	*p*-value
		(95% CI)		(95% CI)		(95% CI)		(95% CI)	
**BMI Trajectory**									
Low	173	21.9	0.03	12.3	<0.01	12.9	<0.01	1.91	<0.01
		(20.8, 23.0)		(10.6, 14.0)		(11.7, 14.1)		(1.81, 2.01)	
High	33	24.2		20.3		19.2		1.49	
		(22.2, 26.2)		(17.3, 23.4)		(17.0, 21.3)		(1.31, 1.67)	
**SUA Trajectory**									
Low	161	22.1	0.64	12.7	0.01	13.5	0.04	1.86	0.11
		(20.9, 23.2)		(10.9, 14.5)		(12.2, 14.8)		(1.75, 1.97)	
High	42	22.5		16.6		15.9		1.72	
		(20.7, 24.3)		(13.7, 19.5)		(13.8, 18.0)		(1.55, 1.88)	
Combined (BMI*SUA) Trajectory									
Low BMI*	137	21.8	0.11	12.0	<0.01	12.8	<0.01	1.91	<0.01
		(20.6, 23.0)		(10.2, 13.8)		(11.5, 14.1)		(1.80, 2.02)	
Low SUA									
Low BMI*	30	21.4		12.4		13.0		1.87	
High SUA		(19.3, 23.4)		(9.3, 15.5)		(10.8, 15.3)		(1.68, 2.06)	
High BMI*	21	23.5		16.5		17.2		1.57	
Low SUA		(21.0, 25.9)		(12.9, 20.2)		(14.6, 19.8)		(1.35, 1.80)	
High BMI*	12	25.2		26.6		22.6		1.35	
High SUA		(22.1, 28.4)		(21.8, 31.4)^a^^,^^b^^,^^c^		(19.1, 26.0)^a^^,^^b^		(1.06, 1.64)^a^^,^^b^	

LS means, Least-squares means; 95% CI, 95% Confidence interval; BMI, Body mass index; SUA, Serum uric acid; AST, Aspartate aminotransferase; ALT, Alanine aminotransferase; γ-GTP, Gamma-glutamyl transferase.

Adjusted for age, sex, maternal educational level and monthly household income at 11–12 years of age.

^a^, Bonferroni-adjusted *p*-value <0.05, compared to the low-low group.

^b^, Bonferroni-adjusted *p*-value <0.05, compared to the low-high group.

^c^, Bonferroni-adjusted *p*-value <0.05, compared to the high-low group.

The third section of Tables 2 and 3 summarizes the combined effect of trajectories of SUA and BMI. The participants were divided into four subgroups (low SUA-low BMI trajectories group [low-risk group]; low SUA-high BMI trajectories group, high SUA-low BMI trajectories group; high SUA-high BMI trajectories group [high-risk group]) to access the combined effect of BMI and SUA trajectories on liver enzymes. When this combination was evaluated with the AST/ALT ratio, the high BMI-high SUA trajectories group showed a lower mean of the AST/ALT ratio. This was significantly lower than the low BMI-low SUA trajectories group and the low BMI-high SUA trajectories group.

**Fig 3:**
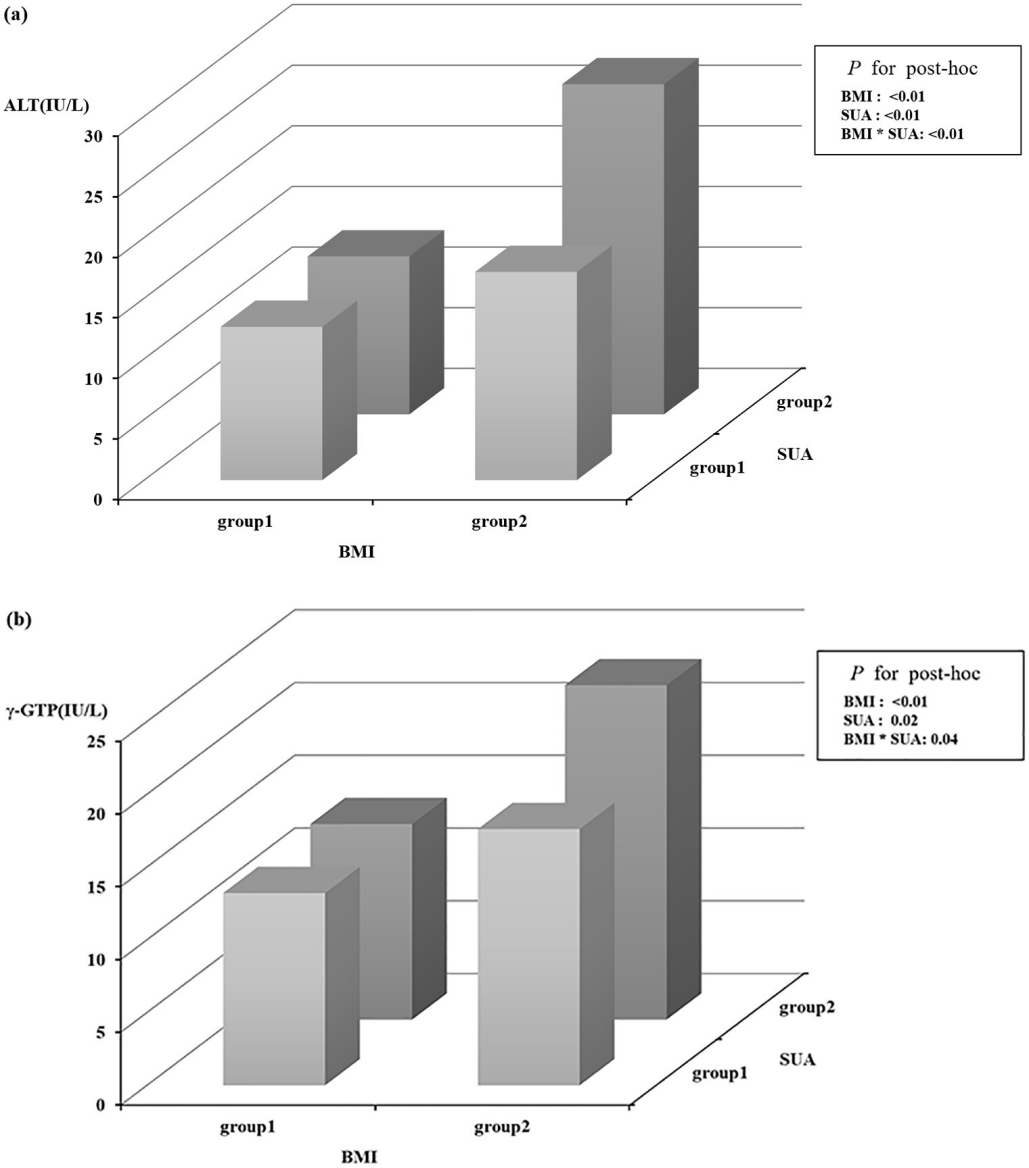
The interactions between SUA trajectory and BMI trajectory on ALT and γ- GTP in adolescent. BMI, Body mass index; SUA, Serum uric acid; Z-score ALT, Alanine aminotransferase; γ-GTP, Gamma-glutamyl transferase. (a) The interactions between SUA trajectory and BMI trajectory on ALT at 11 to 12 years of age; (b) The interactions between SUA trajectory and BMI trajectory on γ-GTP at 11 to 12 years of age;.

Higher mean of ALT and γ–GTP were shown in high-risk group (high SUA-high BMI trajectories group) than in the other groups (Bonferroni-adjusted *p* value <0.05), even when adjusted for sex, age, maternal educational level, and monthly household income. In addition, the interaction of SUA and BMI trajectories reached statistical significance (*p* for interaction <0.01 for ALT and *p* for interaction = 0.04 for γ–GTP). In the high-risk group, ALT and γ–GTP showed greater mean values than when only one risk for BMI or SUA was present, suggesting a synergistic effect (Fig 3). However, AST and AST/ALT ratio did not show a significant interaction effect.

## Discussion

We evaluated SUA and BMI trajectories in childhood have longitudinal association with liver function enzymes in adolescence. The liver enzymes (AST, ALT and γ–GTP) level at 11–12 years of age was higher in high BMI trajectory group than low BMI trajectory group. The AST/ALT ratio was low in the high BMI group, in line with a previous report [26].

High SUA trajectory group had higher mean of liver enzymes especially ALT and γ–GTP than low SUA trajectory group. Combined, relatively high BMI and high SUA trajectories was synergistically associated with elevated liver level of ALT and γ–GTP, respectively. As the high BMI-high SUA group showed a significant mean difference and interaction effects with the other groups for the ALT level, there may have been a threshold effect. Although liver enzyme levels are known to be associated positively with the BMI and SUA level, few studies have involved the evaluation of their interaction. A recent study conducted in China revealed a relationship between the SUA trajectory and new-onset NAFLD, with no significant interaction of abdominal obesity [27].

Elevated plasma concentrations of liver enzyme including AST, ALT, and γ–GTP in children can be a sign of liver inflammation or damage [28]. While Liver enzyme may not be a major factor that directly determines NAFLD, a cross-sectional study conducted on adults reported that elevation of liver enzyme level is a surrogate marker of NALFD [29, 30]. NAFLD is a common chronic liver disease characterized by the accumulation of fat in the liver and is considered as an important feature of metabolic syndrome and an important factor causing cardiovascular diseases [31, 32]. Fatty liver index (FLI) and hepatic steatosis index (HIS) are used as non-invasive biomarkers of hepatic steatosis in numerous epidemiologic studies, which can be calculated from liver enzymes and various metabolic components [33, 34]. These indexes were mostly used for adults, but a single center cross-sectional study from September 2012 to May 2016 indicated that FLI and HSI could be used as noninvasive biomarkers of liver steatosis in Italian pediatric population as well [35]. Recently the prevalence of obesity and NAFLD in children as well as adolescents is increasing fast in globally [36, 37]. Similar to this study, a cross-sectional study conducted in Korea reported that early onset NAFLD and γ–GTP had a significant correlation in school-aged children with obesity [38]. According to a study conducted by an Italian group, the BMI level in children with liver fibrosis was significantly high (27.3±3.8 vs 25.0±3.1, *p* = 0.004) compared to the group without liver fibrosis [39].

SUA promotes oxidative stress and systemic inflammation directly or via lipid and glucose metabolism, leading to hepatocyte death and steatohepatitis [14, 40, 41]. BMI increases are associated with steatosis and NAFLD via inflammation [42, 43]. The mechanisms by which the BMI modifies the association between the SUA level and liver function are unclear, but steatosis and inflammation may be implicated. Additionally, the SUA level is reportedly associated with liver disease, mainly in adults. A study using NHANES in US reported association of SUA with development of chronic liver diseases. Similar to our study, it also reported that SUA level was associated with elevated ALT or γ–GTP [19]. Although SUA not part of any definition of NAFLD, amassing studies have shown that SUA level was significantly associated with NAFLD and elevated SUA level was an independent risk factor for NAFLD in Meta-analysis (RR = 1.03, 95% CI: 1.02–1.05) [44]. Recently a Chinses group identified SUA change patterns via trajectory model, and evaluated their association with new-onset NAFLD. According to the pattern, it was divided into four distinct groups, and the SUA group maintaining a high level was associated with the new-onset of NAFLD (OR 2.34, 95% CI 1.43–3.83) [27]. While a small number of prospective studies focused on association of NAFLD with SUA is being conducted on adults [45], studies on children and adolescents are mostly evaluated as cross-sectional studies.

The result of this study has been shown the same trend as studies conducted by other studies. It was found that the level each of SUA and BMI trajectories from middle to late childhood was significantly associated with liver enzyme level in early adolescence. In this study, the BMI and SUA level independently influenced the ALT and γ-GTP levels and exerted an interaction effect. Nevertheless, SUA and BMI trajectories exhibited a significant association with liver enzyme level in early adolescence, and it was confirmed that the combination of high-high combined trajectory group had a synergetic effect on elevation of ALT and γ–GTP. Moreover, we confirmed that obesity is correlated positively for elevated SUA in children in our previous study [46]. In this study, it was confirmed that the BMI and SUA can independently influence liver function enzymes, and that there is an interaction effect. In addition, the BMI and SUA trajectory groups showed associations with metabolic syndrome components; in addition, the TG level and BP were high and HDL-c level was low in the high BMI trajectory group. The SUA trajectory was associated with the TG level; the high SUA trajectory group had a high TG value.

Therefore, this study is the first study to prospectively observe in BMI and SUA trajectories through a cohort from middle to late childhood in Korea. It is expected that trajectory monitoring of exposure in childhood and early intervention for high-risk group would play a crucial role in preventing progress of related diseases in later stages of life. In addition, as children subject to the study were have not been diagnosed with the relevant disease, further study is required on what additional risk factors are to be considered in progress of liver enzyme level elevation in general children to metabolic syndrome.

The strengths of the present study included a birth cohort data were used to prospectively access causal association between combined trajectories of BMI and SUA on liver enzymes level in general population. Second, trajectory modeling was performed to identify BMI and SUA ranges and patterns of change over time. We used trajectory analysis to examine repeated measures of the BMI and SUA level in the same individuals over time, which enabled the estimation of the longitudinal effects of these variables on liver function [47]. This prospective analysis provided stronger evidence for the associations of the BMI and SUA level with liver function than would cross-sectional analyses. Lastly, as this birth cohort consist of children participants, any effects of risk factors such as alcohol consumption and smoking on liver function were absent. However, some limitations should also be noted. This study included potential selection biases because it was a tertiary-hospital based birth cohort and involved a small sample size. Another limitation was that some measurement errors maybe in play; serum samples were collected only once during each follow-up period. Also, the association between liver enzymes and BMI and SUA trajectories may be underestimated for clinical significance since serum ALT and AST level may also be normal in children and adults with NASH or NAFLD.

In summary, the synergistic effects of BMI and SUA level trajectories on liver enzyme in early adolescence were examined. This study is the first to examine longitudinal associations of the SUA level and BMI with liver enzyme levels in healthy children using trajectory modeling.

This study also emphasized liver enzyme level should be monitored from early life and regulation of SUA level with their obesity would be primary strategy to prevent development related disease in later life.

## Supporting information

S1 Fig The distribution of AST and ALT by BMI and SUA trajectory group. BMI, Body mass index; SUA, Serum uric acid; AST, Aspartate aminotransferase; ALT, Alanine aminotransferase. In group labels, L- means low and H- means high.
(TIF)

S1 File Summary data for Fig 3. The interactions between SUA trajectory and BMI trajectory on ALT and γ- GTP in adolescent. LS means, Least-squares means; 95% CI, 95% Confidence interval; BMI, Body mass index; SUA, Serum uric acid; ALT, Alanine aminotransferase; γ-GTP, Gamma-glutamyl transferase. (a) The interactions between SUA trajectory and BMI trajectory on ALT at 11 to 12 years of age; (b) The interactions between SUA trajectory and BMI trajectory on γ-GTP at 11 to 12 years of age.
(PDF)
