# Supplementary figures and images for: Synergistic effect of serum uric acid and body mass index trajectories during middle to late childhood on elevation of liver enzymes in early adolescence: Findings from the Ewha Birth and Growth Study
Source: PLoS One. 2023 Apr 24;18(4):e0282830. doi: 10.1371/journal.pone.0282830 (PMC10124883; doi:10.1371/journal.pone.0282830)

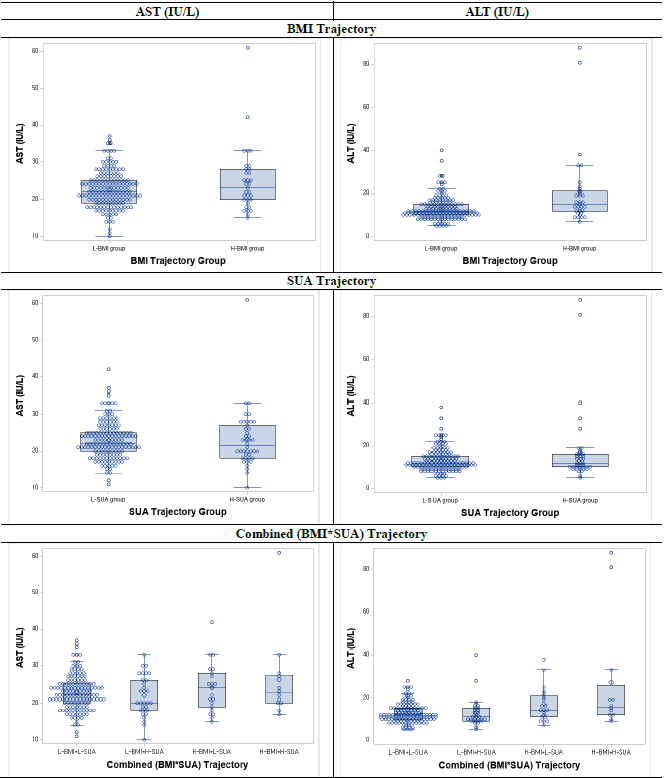

Supplement: S1 Fig — BMI, Body mass index; SUA, Serum uric acid; AST, Aspartate aminotransferase; ALT, Alanine aminotransferase. In group labels, L- means low and H- means high. (TIF) [file pone.0282830.s001.tif]
