# Supplementary material for: Synergistic effect of serum uric acid and body mass index trajectories during middle to late childhood on elevation of liver enzymes in early adolescence: Findings from the Ewha Birth and Growth Study
Source: PLoS One. 2023 Apr 24;18(4):e0282830. doi: 10.1371/journal.pone.0282830 (PMC10124883; doi:10.1371/journal.pone.0282830)
Supplement: S1 File — The interactions between SUA trajectory and BMI trajectory on ALT and γ- GTP in adolescent. LS means, Least-squares means; 95% CI, 95% Confidence interval; BMI, Body mass index; SUA, Serum uric acid; ALT, Alanine aminotransferase; γ-GTP, Gamma-glutamyl transferase. (a) The interactions between SUA trajectory and BMI trajectory on ALT at 11 to 12 years of age; (b) The interactions between SUA trajectory and BMI trajectory on γ-GTP at 11 to 12 years of age. (PDF) [file pone.0282830.s002.pdf]

<Supplemental file>

Summary data for Figure 3. The interactions between SUA trajectory and BMI trajectory on ALT and γ- GTP in adolescent

	n	ALT (IU/L)		γ-GTP (IU/L)	
		**LS Means (95% CI)**	***p*-value**	**LS Means (95% CI)**	***p*-value**
Low BMI* Low SUA	137	12.64 (11.26, 14.01)	<0.01	13.22 (12.22, 14.21)	0.04
Low BMI* High SUA	30	13.02 (10.05, 15.99)		13.44 (11.30, 15.58)	
High BMI* Low SUA	21	17.17 (13.64, 20.70)		17.62 (15.07, 20.17)	
High BMI* High SUA	12	27.22 (22.57, 31.88)		22.98 (19.62, 26.35)[a,b]	

LS means, Least-squares means; 95% CI, 95% Confidence interval; BMI, Body mass index; SUA, Serum uric acid; ALT, Alanine aminotransferase; γ-GTP, Gamma-glutamyl transferase.
(a) The interactions between SUA trajectory and BMI trajectory on ALT at 11 to 12 years of age; (b) The interactions between SUA trajectory and BMI trajectory on γ-GTP at 11 to 12 years of age;
